# Adult-Onset Asthma Associated With E-Cigarette Use

**DOI:** 10.7759/cureus.19190

**Published:** 2021-11-01

**Authors:** Jessica Roberts, Joni Chow, Kovid Trivedi

**Affiliations:** 1 Family Medicine, Yakima Valley Farm Workers Clinic, Grandview, USA; 2 Pediatrics, College of Osteopathic Medicine of the Pacific Northwest, Lebanon, USA; 3 Pulmonary/Critical Care Medicine, Salem Pulmonary Associates/Salem Health, Salem, USA

**Keywords:** vaping, e-cigarettes, electronic cigarettes, nicotine addiction, cigarette smoking, corticosteroids, refractory asthma

## Abstract

Electronic cigarettes (e-cigarettes) are being increasingly used as a “safer” alternative to regular cigarettes as a method of de-addiction or a bridge to nicotine cessation. However, a multitude of pulmonary pathologies have been described associated with its use and have been clubbed under the category of e-cigarette or vaping use-associated lung injury (EVALI). This case describes a patient who started e-cigarette smoking in order to quit combustible cigarette smoking and developed adult-onset severe asthma. The clinical effect was initially reversible but later developed into persistent symptoms requiring inhaled and systemic therapy.

## Introduction

Electronic cigarettes (e-cigarettes) have become a mainstay in today’s culture with many smokers utilizing them in their pursuit of smoking cessation or a “safer” alternative to traditional combustible cigarettes. E-cigarettes offer flexibility on the quantity of nicotine being used. The safety of e-cigarettes was called into question by the numerous cases of e-cigarette or vaping use-associated lung injury (EVALI), some of which were fatal. These cases highlighted the reality that e-cigarettes may not be the benign alternative to cigarettes that many were seeking [1]. While EVALI is a severe complication of using e-cigarettes, induction or worsening asthma symptoms are far more prevalent in e-cigarette users. Adult-onset asthma can be induced by different triggers, which cause inflammation leading to acute bronchoconstriction [2]. Symptoms of asthma include wheezing, chest tightness, cough, mucus hypersecretion, and shortness of breath. Improvement of forced expiratory volume (FEV1) and/or forced vital capacity (FVC) after administration of bronchodilators can also be seen [3]. It is long known that cigarette smoke is a major trigger of asthma exacerbations, which may be one reason why some smokers made the switch to e-cigarettes. Studies, however, have shown that while e-cigarette liquid may be nicotine-free and does not contain the same toxic chemicals found in traditional combustible cigarettes, it still contains multiple toxins and irritants, which have been connected to new and worsening lung function [4]. This case report describes a case of adult-onset severe asthma in a life-long cigarette smoker who had recently begun using e-cigarettes for cigarette smoking cessation.

## Case presentation

A 59-year-old female was referred to the pulmonary clinic for evaluation of wheezing, shortness of breath with exertion, and cough ongoing for the last five months. She was apparently asymptomatic before that. She denied chest pain, orthopnea, leg swelling, heartburn, sinus drainage, known environmental allergies, or any other complaints. The patient admitted to gaining 5 pounds (Lbs) of weight since the symptoms started (BMI 34.9). She started smoking cigarettes at the age of 16 and had smoked one1 pack per day since then till about six months ago (42 pack-years) when she started smoking e-cigarettes in a successful attempt to quit cigarette smoking. Incidentally, her partner continued to smoke cigarettes in the house. The patient’s indoor environment also consisted of three dogs and three cats, which she has had for at least five years. No indoor plants. She used to work as a beauty technician and had retired about a year back. No previous history of asthma or allergies. No family history of asthma or other lung-related ailments. No history of other significant organic or inorganic material exposures. The patient’s past medical history was significant for hypothyroidism, Asperger’s syndrome, Type II diabetes mellitus, peripheral neuropathy, dyslipidemia, endometriosis, and hypertension. The patient was diagnosed with asthma/chronic obstructive pulmonary disease (COPD) by her primary care physician and started on a tiotropium inhaler 18 mcg inhalation daily, beclomethasone 80 mcg inhaler, two puffs twice daily, and albuterol inhaler as needed for breakthrough symptoms. The patient was found eligible and enrolled in the lung cancer screening program. Her low dose computerized tomography scan of the chest (LDCT chest) showed mild emphysematous changes with no lung nodules. Pulmonary function testing (PFT) results are displayed in Table 1. The patient was diagnosed with new-onset asthma, likely induced by the initiation of e-cigarettes and uncontrolled at the time of evaluation.

 

**Table 1:** Pulmonary Function Testing (PFT) FEV1: Forced expiratory volume in one second; FVC: Forced vital capacity; TLC: Total lung capacity; RV: Residual volume; ERV: Expiratory reserve volume

	Pre-bronchodilator	Post-bronchodilator
FEV1/FVC	72%	78%
FEV1	58% (1.50 L)	66% (1.70 L)
FVC	62% (2.07 L)	66% (2.18 L)
TLC	76% (3.71 L)	
RV	89% (1.64 L)	
ERV	20% (0.20 L)	

Upon evaluation in the Pulmonary Clinic, her treatment regimen was optimized to include high dose inhaled corticosteroids (ICS), inhaled long-acting beta-agonists (LABA), inhaled long-acting muscarinic antagonists (LAMA), and short-acting inhaled beta-agonists (SABA). She was advised to discontinue e-cigarette use immediately and was started on nicotine patches as replacement therapy.

At the one month follow-up clinic visit, the patient said that she had quit smoking e-cigarettes but had relapsed into conventional cigarette smoking. She denied the use of nicotine patches. Her symptoms were persistent despite new inhaler therapy after the last visit, which led her to quit e-cigarette use. After cessation, her symptoms had completely resolved. She was advised to continue regular use of the current asthma regimen till the next visit, where a step-down of therapy would be started if symptoms remained well controlled. She was counseled on smoking cessation.

The patient missed her next follow-up visit and came back to the clinic five months later. She had restarted e-cigarette smoking. She complained of worsening respiratory symptoms despite being on the same bronchodilator regimen. ICS dose was increased, and the patient was given a taper of oral corticosteroid as a bridge. Counseling was provided to quit e-cigarette use using nicotine patches and behavioral therapy.

Over the course of the next two years, montelukast and fluticasone nasal spray were added for asthma control in addition to inhaled therapies and several courses of oral corticosteroid. The patient had missed clinic appointments a number of times. For further workup, cardiac etiologies of dyspnea were ruled out. Sleep evaluation was done and the patient was started on bilevel positive airway pressure (BiPAP) therapy for obstructive sleep apnea and hypoventilation syndrome. Weight had fluctuated within 10 Lbs. The patient switched from e-cigarettes to cigarette smoking again and the symptoms did not improve this time. Follow-up PFT did not show any significant change. Serum allergen panel targeted towards common local allergens showed Immunoglobulin E (IgE) response to *Aspergillus fumigatus*, and alder and birch trees. Total IgE was elevated at 615 kU/L. The absolute eosinophil count in peripheral blood was 210/ml. Due to persistent symptoms with multiple exacerbations requiring oral corticosteroid (OCS), the patient was started on scheduled OCS with a plan to switch to a biologic agent pending insurance approval. Unfortunately, she still continues to smoke cigarettes despite numerous attempts at smoking cessation with various modalities. 

## Discussion

Despite the rising popularity of e-cigarettes as a commonly perceived safer alternative to tobacco cigarettes, the risks of harmful outcomes are not fully recognized by the majority of the population. Numerous studies have suggested that the use of e-liquid, with or without nicotine, decreases lung function, most likely due to the toxins and irritants found in the liquid itself. Furthermore, the metal coils used in the heating and cooling process of the e-liquid are a source of chromium, manganese, nickel, and lead, ultimately emitted as metallic nanoparticles and then inhaled [5]. In our case, the patient, who had a history of chronic tobacco use, did not develop asthma symptoms until she began using e-cigarettes. This suggests that the compounds found specifically in the e-cigarette are irritating enough to induce asthma in patients without a known asthma diagnosis. Initially, her symptoms had resolved on cessation of e-cigarette use, but later, after reuse of e-cigarettes, even cessation did not improve symptoms indicating long-lasting effects from exposure. One study reported that inhalation of e-liquid increases pro-inflammatory mediators, such as IL-10 and TNF-α which induce acute bronchoconstriction even after just five minutes of use [6]. E-cigarettes remain popular despite warnings of decreased lung function and EVALI, prompting the CDC to issue an official health advisory on it [7]. Research focused on assessing the long- and short-term damage of e-cigarettes is still in its infancy. Our case further supports the risks of utilizing e-cigarettes and, hopefully, future studies will provide further education about the harmful outcomes of e-cigarettes and e-liquid. 

## Conclusions

The use of e-cigarettes is not a safer alternative to combustible cigarette smoking and can lead to short and long-term implications, some of which can be refractory to conventional therapies. Our patient developed adult-onset asthma likely as a result of e-cigarette use, which initially was reversible, but later transformed into a refractory process requiring systemic corticosteroid use in addition to conventional inhaled therapies. 

## References

[1] Clapp PW, Peden DB, Jaspers I (2020). E-cigarettes, vaping-related pulmonary illnesses, and asthma: a perspective from inhalation toxicologists. J Allergy Clin Immunol.

[2] Tillie-Leblond I, Gosset P, Tonnel AB (2005). Inflammatory events in severe acute asthma. Allergy.

[3] Abramson MJ, Perret JL, Dharmage SC, McDonald VM, McDonald CF (2014). Distinguishing adult-onset asthma from COPD: a review and a new approach. Int J Chron Obstruct Pulmon Dis.

[4] Clapp PW, Jaspers I (2017). Electronic cigarettes: their constituents and potential links to asthma. Curr Allergy Asthma Rep.

[5] Chun LF, Moazed F, Calfee CS, Matthay MA, Gotts JE (2017). Pulmonary toxicity of e-cigarettes. Am J Physiol Lung Cell Mol Physiol.

[6] Kotoulas SC, Pataka A, Domvri K (2020). Acute effects of e-cigarette vaping on pulmonary function and airway inflammation in healthy individuals and in patients with asthma. Respirology.

[7] Adhikari R, Koritala T, Gotur R, Malayala SV, Jain NK (2021). EVALI - e-cigarette or vaping product use-associated lung injury: a case report. Cureus.

